# Influence of Visual Deprivation on Auditory Spectral Resolution, Temporal Resolution, and Speech Perception

**DOI:** 10.3389/fnins.2019.01200

**Published:** 2019-11-06

**Authors:** Hyun Joon Shim, Geurim Go, Heirim Lee, Sung Won Choi, Jong Ho Won

**Affiliations:** ^1^Department of Otorhinolaryngology-Head and Neck Surgery, Eulji Medical Center, Eulji University School of Medicine, Seoul, South Korea; ^2^Department of Psychology, Duksung Women’s University, Seoul, South Korea; ^3^Division of ENT, Sleep Disordered Breathing, Respiratory, and Anesthesia, Office of Product Evaluation and Quality, Center for Devices and Radiological Health, U.S. Food and Drug Administration, Silver Spring, MD, United States

**Keywords:** early blindness, late blindness, visual deprivation, spectral resolution, temporal resolution, speech perception

## Abstract

We evaluated whether blind subjects have advantages in auditory spectral resolution, temporal resolution, and speech perception in noise compared with sighted subjects. We also compared psychoacoustic performance between early blind (EB) subjects and late blind (LB) subjects. Nineteen EB subjects, 16 LB subjects, and 20 sighted individuals were enrolled. All subjects were right-handed with normal and symmetric hearing thresholds and without cognitive impairments. Three psychoacoustic measurements of the subjects’ right ears were performed via an inserted earphone to determine spectral-ripple discrimination (SRD), temporal modulation detection (TMD), and speech recognition threshold (SRT) in noisy conditions. Acoustic change complex (ACC) responses were recorded during passive listening to standard ripple-inverted ripple stimuli. EB subjects exhibited better SRD than did LB (*p* = 0.020) and sighted (*p* = 0.003) subjects. TMD was better in EB (*p* < 0.001) and LB (*p* = 0.007) subjects compared with sighted subjects. SRD was positively correlated with the duration of blindness (*r* = 0.386, *p* = 0.024). Acoustic change complex data for ripple noise change at the Cz and Fz electrodes showed trends toward significant correlations with the behavioral results. In conclusion, compared with sighted subjects, EB subjects showed advantages in terms of auditory spectral and temporal resolution, while LB subjects showed an advantage in temporal resolution exclusively. These findings suggest that it might take longer for auditory spectral resolution to functionally enhance following visual deprivation compared to temporal resolution. Alternatively, a critical period of very young age may be required for auditory spectral resolution to improve following visual deprivation.

## Introduction

Humans commonly perceive surrounding information via sensory convergence. The integration of multiple senses can reduce perceptual ambiguity, speed up reactions, and increase the accuracy of stimulus detection. Because blind subjects only use auditory signals to communicate, without lip reading, investigating their speech perception ability compared with sighted subjects is an important aspect of developing rehabilitation programs. However, the influence of visual deprivation on speech recognition in real life remains unclear. Few studies have compared speech perception without visual cues between blind and sighted subjects, and the results varied considerably (Muchnik et al., 1991; Hugdahl et al., 2004; Gougoux et al., 2009; Arnaud et al., 2018). This variability may be due to the small sample sizes, inclusion of mixtures of subjects with congenital and acquired blindness, and the inclusion of some subjects with residual vision. Therefore, in the current study, we enrolled a relatively large number of young adults with EB and LB as separate groups if they met World Health Organization blindness categories 4 and 5 (Vashist et al., 2017) to compare speech perception abilities among early blind (EB) subjects, late blind (LB) subjects, and sighted controls. Auditory spectral resolution and temporal resolution are fundamental aspects of speech perception. Therefore, investigating the influence of visual deprivation on those factors could help us to understanding the potential changes in speech processing induced by visual deprivation. Here, we evaluated three psychoacoustic performance measures (i.e., spectral resolution, temporal resolution, and speech perception in noise) in each group. Although it has been reported that compared with sighted subjects, blind subjects show better spectral and temporal resolutions, as measured by pitch discrimination or pure-tone discrimination (Gougoux et al., 2004; Rokem and Ahissar, 2009; Wan et al., 2010; Voss and Zatorre, 2012) and an auditory backward temporal masking task (Stevens and Weaver, 2005), the acoustic stimuli used in these previous studies were often band-limited and, therefore, may not reflect the temporal and spectral resolution of hearing for broad-band stimuli such as speech signals. Thus, in the current study, we used a broad-band noise carrier that is either spectrally modulated (i.e., rippled spectra noise to evaluate spectral envelope sensitivity) or temporally modulated (i.e., temporally modulated noise to evaluate amplitude envelope sensitivity).

Based on behavioral and neurophysiological findings, two different views have been proposed in the literature that attempted to explain the auditory performance of blind subjects (Pascual-Leone et al., 2005). The first view, called compensation theory, proposes that visual cortices of blind subjects may be recruited to perform auditory functions in a compensatory cross-modal manner, so blind subjects may show better performance compared to sighted subjects. Indeed, several studies have demonstrated advantages of blind subjects over sighted subjects in auditory memory (Amedi et al., 2003), pitch discrimination (Gougoux et al., 2004; Wan et al., 2010), sound localization (Röder et al., 1999; Fieger et al., 2006), dichotic listening (Hugdahl et al., 2004), and ultra-fast speech comprehension (Moos and Trouvain, 2007), supporting the compensation theory. The second view, called general-loss theory, proposes that vision is inevitable to develop certain auditory performances in particular with regards to auditory sense of space, and as a result, blind subjects are impaired in spatial tasks (Zwiers et al., 2001; Gori et al., 2014; Finocchietti et al., 2015; Cappagli et al., 2017b). The current study was designed based upon the compensation theory because the acoustic stimuli used in the current study did not present any binaural auditory cues. Therefore, our first hypothesis in the present study was that blind subjects may show better psychoacoustic performance than sighted subjects, because of the recruitment effect of visual cortices in blind subjects.

Some studies showed that EB subjects had advantages over LB subjects in terms of pitch discrimination (Gougoux et al., 2004; Wan et al., 2010), melody discrimination (Voss and Zatorre, 2012), and sound source discrimination (Voss et al., 2008), and two of those studies reported that their pitch discrimination (Gougoux et al., 2004) and sound source discrimination (Voss et al., 2008) were better in people who developed blindness earlier in life. In line with these data, other studies reported positive correlations of the duration of blindness with short-range functional connectivity density in the primary visual cortex (Qin et al., 2015) and blood oxygenation level dependent responses in the left middle occipital gyrus (Tao et al., 2015). These findings support the compensation theory and suggest that there is a critical period for cross-modal reorganization of brain and functional enhancement, or enough duration of blindness may be required for compensatory change of auditory cortex. However, other studies reported that LB subjects had better haptic orientation perception (Postma et al., 2008), auditory distance discrimination and proprioceptive reproduction (Cappagli et al., 2017a), and dynamic audio localization (Finocchietti et al., 2015) compared with EB subjects. The duration of blindness in LB subjects was negatively correlated with performance on a spatial bisection task (Amadeo et al., 2019), sound-related activity in the occipital cortex (Collignon et al., 2013; Amadeo et al., 2019), and the white matter fractional anisotropy in the corpus callosum (Wang et al., 2013). The studies referenced above support the second view (general-loss theory) that vision is the only sense to encode spatial information and it cannot be replaced by other sensory modalities. We speculated spectral resolution, temporal resolution, and speech perception without any binaural cues may support the compensation theory which has been demonstrated by numerous previous studies for sound perception such as pitch discrimination rather than the general-loss theory for spatial task. Therefore, our second hypothesis was that EB subjects may show better psychoacoustic performances compared with LB subjects.

To test both of our hypotheses, we compared the auditory spectral resolution, temporal resolution, and speech perception among EB subjects, LB subjects, and sighted controls, and determined correlations between these psychoacoustic performance measures and the duration of blindness and age at blindness onset.

The acoustic change complex (ACC) is an auditory evoked potential (P1-N1-P2) that can be elicited by the listener’s ability to detect a change in an ongoing sound in passive listening conditions (Martin and Boothroyd, 1999; Tremblay et al., 2003). ACC can be measured as a spectral change within an ongoing ripple noise stimulus (Won et al., 2011a). Therefore, in addition to the behavioral psychoacoustic experiments, we also recorded the ACC in response to standard ripple-inverted ripple stimuli to characterize the central processing of spectral information of sound in each group.

## Materials and Methods

### Subjects

We included 19 EB subjects (28.8 ± 8.0 years, male:female [M:F] = 11:8), 16 LB subjects (33.4 ± 5.9 years, M:F = 7:9), and 20 sighted subjects (25.4 ± 3.9 years, M:F = 10:10). There were significant differences in age among the three groups [H(2) = 10.589; *p* = 0.005] and the Mann–Whitney *post hoc* analysis revealed that the sighted group was younger than the LB group (*U* = 48.000; *z* = –3.577; *p* < 0.001; Table 1). All of the subjects were right-handed, aged <40 years, had normal and symmetric hearing thresholds (≤20 dB hearing level at 0.25, 0.5, 1, 2, 3, 4, and 8 kHz), and had no neurological deficits or cognitive impairments. For the EB group, we recruited blind subjects who were born blind or became blind at birth or within a few days of birth. The mean duration of blindness in EB subjects was 28.8 ± 8.0 years. For the LB group, we recruited blind subjects who lost their sight at ≥9 years old and their mean age of onset was 20.8 ± 7.4 years (range 9–32 years) with a variance of 55.4 (Table 2). The mean duration of blindness in the LB subjects was 12.6 ± 6.3 years (range: 5–28 years). The duration of blindness was significantly shorter in LB subjects than in EB subjects (*U* = 14.500; *z* = –4.561; *p* < 0.001). The pure tone averages were not significantly different among three groups. Table 1 presents the demographic and audiologic characteristics of the three groups, and Table 2 presents the characteristics of blind subjects. This study was carried out in accordance with the Declaration of Helsinki and the recommendations of the Institutional Review Board of Eulji Medical Center with written informed consent from all subjects. The informed consent form was verbally presented to the blind subjects in the presence of impartial witnesses, and they then signed the form and a copy was given to them.

**TABLE 1:** Comparison of age, sex, and pure-tone average among early blindness group, late blindness group and sighted control group.

	**Early blindness (*N* = 19)**	**Late blindness (*N* = 16)**	**Sighted subjects (*N* = 20)**	***p***
Age (year)	28.8 ± 8.0	33.4 ± 5.9	25.4 ± 3.9	0.005
Sex (M/F)	11:8	7:9	10:10	ns
Blindness duration (year)	28.8 ± 8.0	12.6 ± 6.3		*p* < 0.001
Mean PTA, right (dB hearing level)	4.67 ± 3.22	6.41 ± 4.03	5.31 ± 3.22	ns
Mean PTA, left (dB hearing level)	5.72 ± 3.54	6.95 ± 4.98	4.75 ± 3.13	ns

*PTA indicates pure-tone average; ns, not significant (*p* > 0.05). The Kruskal–Wallis test was used to compare age and mean PTA. The χ^2^ test was used to compare the sex distribution. The Mann–Whitney test was used to compare the blindness duration.*

**TABLE 2:** Characteristics of blind subjects.

**Subject**	**Age (yeas)**	**Age of onset (years)**	**Gender**	**Handed-ness**	**Visual acuity**	**Cause of blindness**
EB-1	32	Birth	M	Right	No light perception	Optic nerve atrophy
EB-2	19	Birth	M	Right	No light perception	Optic nerve atrophy
EB-3	36	Birth	F	Right	No light perception	Congenital cataract
EB-4	38	Birth	F	Right	Light perception	Retinitis pigmentosa
EB-5	36	Birth	M	Right	Hand motion	Retinitis pigmentosa
EB-6	19	Birth	F	Right	Hand motion	Optic nerve atrophy
EB-7	21	Birth	M	Right	Light perception	Retinopathy of prematurity
EB-8	36	Birth	M	Right	Light perception	Optic nerve atrophy
EB-9	21	Birth	F	Right	Hand motion	Microphthalmos
EB-10	22	Birth	M	Right	Light perception	Leber hereditary optic neuropathy
EB-11	39	Birth	F	Right	No light perception	Optic nerve atrophy
EB-12	37	Birth	F	Right	Light perception	Retinitis pigmentosa
EB-13	39	Birth	M	Right	Light perception	Congenital glaucoma
EB-14	27	Birth	M	Right	Hand motion	Congenital glaucoma
EB-15	23	Birth	M	Right	Light perception	Microphthalmos
EB-16	21	Birth	M	Right	No light perception	Birth trauma
EB-17	37	Birth	M	Right	No light perception	Optic nerve atrophy
EB-18	19	Birth	F	Right	No light perception	Congenital glaucoma
EB-19	26	Birth	F	Right	Light perception	Corneal opacity
LB-1	37	32	F	Right	Light perception	Traumatic maculopathy
LB-2	35	25	M	Right	No light perception	Retinal detachment
LB-3	39	32	M	Right	Hand motion	Retinitis pigmentosa
LB-4	25	15	M	Right	Hand motion	Corneal opacity and retinopathy
LB-5	24	16	M	Right	Hand motion	Leber hereditary optic neuropathy
LB-6	37	9	M	Right	No light perception	Secondary glaucoma
LB-7	38	18	F	Right	Hand motion	Retinitis pigmentosa
LB-8	27	10	F	Right	Hand motion	Neuromyelitis optica
LB-9	36	30	F	Right	Hand motion	Retinitis pigmentosa
LB-10	36	19	F	Right	Hand motion	Retinitis pigmentosa
LB-11	39	23	F	Right	Hand motion	Retinitis pigmentosa
LB-12	35	16	F	Right	No light perception	Corneal opacity and secondary glaucoma
LB-13	21	13	F	Right	No light perception	Morning glory syndrome
LB-14	33	26	M	Right	Hand motion	Retinitis pigmentosa
LB-15	33	21	M	Right	Hand motion	Leber hereditary optic neuropathy
LB-16	39	27	F	Right	Hand motion	Retinitis pigmentosa

*EB, indicates early blindness; LB, late blindness.*

### Behavioral Psychoacoustic Tests

Spectral-ripple discrimination (SRD), temporal modulation detection (TMD), and speech recognition threshold (SRT) in noise were assessed. The SRD test evaluated spectral resolution by measuring the ability of the participants to discriminate a reversal in the phase of a ripple shape. TMD was used to evaluate the listener’s sensitivity to the temporal envelope by discriminating modulated noise from steady noise. All tests were conducted in a sound-attenuating booth. The stimuli were presented monaurally to the right ear via an inserted earphone (Etymotic, ER-3A) at a sampling frequency of 44,100 Hz using a customized MATLAB program.

### Spectral-Ripple Discrimination (SRD) Test

The SRD test was performed as described by Won et al. (2007). To create spectral-ripple stimuli, the following equation was used:

(1) $$s\left(t\right)=\sum_{i=1}^{2555}10^{D\times \left\{a\,b\,s\,\left[\sin\left(\pi \times R\times F_{i}+\emptyset \right)\right]\right\}/20}\times \sin\left(2\times \pi \times 50\times 100^{\frac{i-1}{2555}}\times t+\varphi_{i}\right)$$

in which *D* is the ripple depth in dB, *R* is ripples/octave, *F*_*i*_ is the number of octaves at the *i*th component frequency (i.e.,[(*i*−1)*log*_10_⁡(50)]/[200*log*_10_⁡(2)]), φis the spectral modulation starting phase in radians, *t* is time in seconds, *φ*_1_ is the randomized phase in radians (ranging between 0 to 2π) for each of the 2,555 pure-tone components. A ripple depth (*D*) of 30 dB was used. For the reference stimulus, the spectral modulation starting phase of the full-wave-rectified sinusoidal spectral envelope was set to zero radians, and for the “oddball” stimulus, the phase was set to π/2 radians. The pure tones were spaced equally on a logarithmic frequency scale with a bandwidth of 100–4991 Hz, ensuring a clear representation of the spectral peaks and valleys for stimuli with higher ripple densities. The ripple peaks were spaced equally on a logarithmic frequency scale. The stimuli had a total duration of 500 ms and were ramped with 150 ms linear rise/fall times. The stimuli were filtered with a long-term, speech-shaped filter that was created in CoolEdit 2000, with parameters specified in accordance with the findings of Byrne et al. (1994). The stimuli were presented at 65 dBA. To measure SRD thresholds, a three-interval, three-alternative forced-choice (3-AFC) paradigm with an adaptive two-up and one-down procedure was used. The order of presentation of the three tokens was randomized, and the subject’s task was to select the “oddball” stimulus. A custom-designed clicker was used for both sighted and blind subjects. The subjects were instructed to press the button associated with the order of the oddball stimulus that was presented. For example, if subjects thought that the second sound was the oddball, they were instructed to press the second button of the clicker. The ripple density was varied between 0.125 and 11.314 ripples per octave in equal-ratio steps of 1.414 in an adaptive manner with 13 reversals that converges to the 70.7% correct point (Levitt, 1971). A level attenuation of 1–8 dB (in 1-dB increments) was randomly selected for each interval in the three-interval task. The SRD threshold for each adaptive run was calculated as the geometric mean of the last eight reversals of 13 reversals. The SRD threshold was determined by averaging the threshold from three testing runs.

### Temporal Modulation Detection (TMD) Test

The TMD test was performed using the method described by Won et al. (2011b). For the modulated stimuli, sinusoidal amplitude modulation was applied to a fresh wideband white noise carrier for each presentation. A modulation frequency of 100 Hz was used. The stimuli were presented at 65 dBA. The stimulus duration for both modulated and steady signals was 1 s. The modulated and steady signals were gated on and off with 10-ms linear ramps and they were concatenated with no gap between the two signals. The TMD threshold was measured using a 2-interval, 2-AFC paradigm. One of the intervals contained modulated noise, and the other interval consisted of steady noise. The subjects were asked to press either the first or second button that was associated with the order of the presentation of modulated noise. For example, if the subjects thought that the second sound was modulated noise, they were instructed to press the second button of the clicker. A two-down, one-up adaptive procedure was used to measure TMD threshold. The TMD thresholds (in dB) relative to 100% modulation [i.e., 20log_10_ (*m*_*i*_)] were obtained, where *m*_*i*_ indicates the modulation index. The adaptive tracking procedure began with a modulation depth of 100% and changed in steps of 4 dB from the first to the fourth reversal, and 2 dB for the next 10 reversals. The TMD threshold for each adaptive test run was calculated as the mean of the final 10 reversals. The TMD threshold was determined by averaging the thresholds from three separate test runs.

### Speech Recognition Threshold (SRT) Test

To measure SRTs, equally difficult spondee words in Korean (two syllable words that are both pronounced with equal stress) spoken by a male speaker were presented in noise, which was spectrally shaped to have the same long-term power spectrum as the spondees. In all trials, the masker was gated on and off with 50-ms linear ramps 500 ms before and 50 ms after the target spondees. The mixture of the target spondee and masker stimuli was presented monaurally to the test ear. SRTs corresponding to 50% intelligibility were measured using a one-up, one-down adaptive procedure. Each run started with a signal-to-noise ratio (SNR) of 6 dB, for which subjects were easily able to identify the spondee correctly. The subjects were instructed to repeat the spondee word that they heard. The tester then determined if the subjects repeated the spondee word correctly. If a subject correctly repeated the spondee, the SNR for the next spondee was decreased; otherwise, the SNR was increased. The level of the target spondee was fixed at 65 dBA and the level of the noise was varied in an adaptive manner. An initial step size of 4 dB was used for the first two reversals in the adaptive track, after which the step size was fixed at 2 dB for the next six reversals. When a subject showed a total 8 reversals, the adaptive run ended. The SRT for a given run was based on the average of the SNRs at each of the last six reversals of 8 reversals in the adaptive track. No spondee was repeated for any subject. Three adaptive runs were completed. The final SRT for each subject was taken as the mean of three separate adaptive runs.

### Electrophysiological Methods

#### Acoustic Change Complex (ACC) Stimuli

**FIGURE 1:**
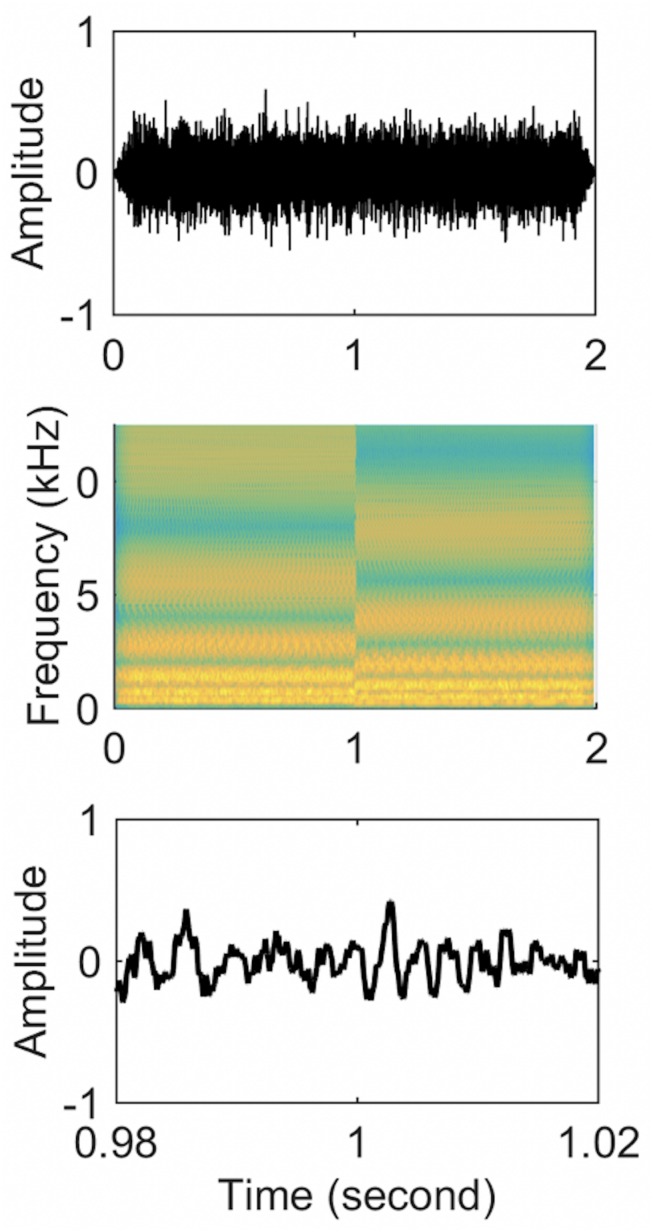
Stimuli waveforms **(upper panel)**, spectrograms **(middle panel)**, and zoom-in of time-domain waveforms from 0.98 to 1.02 s **(lower panel)** for a standard-inverted stimulus. The ripple density was 1 ripple/octave for both stimuli.

The first 1 s of the stimulus consisted of a standard ripple and the last 1 s contained an inverted ripple. Therefore, there was a spectral change at the 1-s point. Each half of each stimulus was individually created and then concatenated (Figure 1). Because the concatenation could produce frequency components beyond 5 kHz at the 1-s point, a 5 kHz low-pass filter was applied to the entire 2-s stimuli. To prevent subjects from perceiving a cue due to the temporal pattern of transition from the standard to inverted ripple at the 1-s point, the phase of the first and last 1 s was randomized for each presentation using random phases for the 2555 individual frequency components. Four ripple density conditions were tested (0.25, 1, 4, and 8 ripples/octave). The 2-s duration stimuli were presented with an inter-stimulus interval of 2 s. Each ripple density condition involved 100 random presentations of standard ripple-inverted ripple stimuli.

#### Procedure

Acoustic change complex responses were recorded across 32 channels using the actiCHamp Brain Products recording system (Brain Products GmbH, Inc., Munich, Germany) during passive listening to standard ripple-inverted ripple stimuli. The blind subjects sat in a comfortable chair reading a braille book. During recording, we encouraged the participants to stay still during the test with their heads and the elbows within a fixed range. We also instructed them not to move wrist and finger. The sighted subjects sat watching a muted, close-captioned movie. A notch filter at 60 Hz was set to prevent powerline noise and the impedance of all scalp electrodes was kept <5 kΩ.

#### Data Processing

The collected data were analyzed using Brain vision analyzer version 2.0 (Brain Products GmbH, Inc). The band-pass filter was set at 0.1∼60 Hz after removing eye blinks and body movement artifacts. In addition, independent component analysis was used to adjust for eye blinks. Ripple stimuli data were separated from 200 ms before stimulus presentation to 200 ms after stimulation (−200 to 2200 ms) based on the standard ripple stimulus presentation time. Baseline correction was performed using the interval before stimulation presentation, and potential averaging was performed. Using a semi-automatic peak detection algorithm in the Brain vision analyzer software, the largest negative deflection that occurred between 100 and 200 ms after stimulus onset was defined as the peak amplitude of N1. The peaks were visually inspected and manually adjusted if necessary.

### Statistical Analysis

The younger age of sighted subjects compared with the LB subjects might have affected the results because sighted subjects had less experience compared to LB subjects. To control for differences in age among the groups, analysis of covariance (ANCOVA) with the Bonferroni *post hoc* test was used to compare the SRD thresholds, TMD thresholds, and peak amplitude of N1 among the three groups. The Kruskal–Wallis test with Bonferroni’s correction (α = 0.05/3 = 0.017) was used to compare age and SRT. Correlation analyses were determined using Pearson’s correlation coefficient.

## Results

**FIGURE 2:**
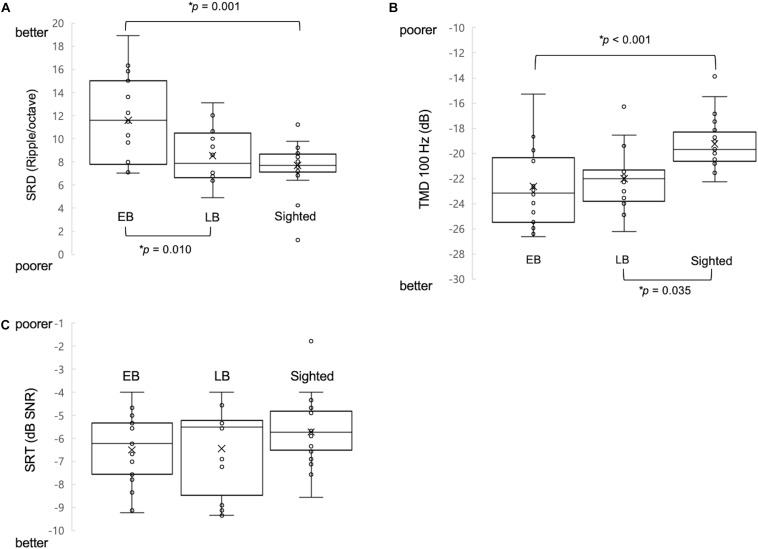
Comparison of three measures of psychoacoustic performance and acoustic change complex (ACC) among early blind (EB), late blind (LB), and sighted subjects. **(A)** The spectral-ripple discrimination (SRD) threshold was greater in EB subjects (i.e., better SRD) than in LB and sighted subjects. **(B)** The temporal modulation detection (TMD) threshold was lower (i.e., better TMD) in EB subjects and in LB subjects compared with sighted subjects. **(C)** The speech reception thresholds (SRTs) in noise were not significantly different among the three groups of subjects. ^∗^Significantly different between the indicated groups (*p* < 0.05).

**FIGURE 3:**
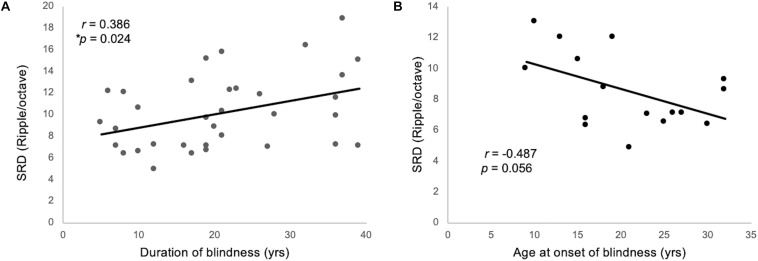
**(A)** The SRD threshold was significantly correlated with the duration blindness duration among 35 individuals from the EB and LB groups. **(B)** For 16 LB subjects, SRD thresholds showed a trend toward significance in the correlation with the ages of onset of blindness.

**FIGURE 4:**
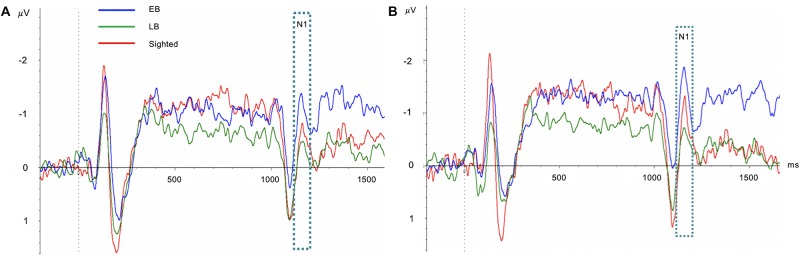
Comparisons of acoustic change complex (ACC) at the Cz **(A)** and Fz **(B)** electrodes among early blind (EB), late blind (LB), and sighted subjects. Although the N1 peak amplitude originating from the inverted ripple sound was numerically greater in EB subjects than in LB subjects or sighted subjects, there were no statistically significant differences.

One-way ANCOVA controlling for age revealed a significant difference in the SRD thresholds among the EB subjects, LB subjects, and sighted subjects (11.6 ± 3.6, 8.6 ± 2.4, and 8.0 ± 1.7 ripples/octave, respectively, *F*(2:49) = 9.116; *p* < 0.001). The Bonferroni *post hoc* test revealed that the SRD threshold was significantly better in EB subjects than in LB subjects and sighted subjects (*p* = 0.010 and *p* = 0.001, respectively; Figure 2A). One-way ANCOVA also revealed a significant difference in the TMD thresholds among the three groups (−22.6 ± 3.1, −22.0 ± 2.4, and −18.8 ± 2.2 dB, respectively, *F*(2:51) = 8.980; *p* < 0.001). The Bonferroni *post hoc* test revealed that TMD was better in EB and LB subjects than in sighted subjects (*p* < 0.001 and *p* = 0.035, respectively; Figure 2B). There was no significant difference in the SRT in noise among three groups [H(2) = 1.478; *p* = 0.478; Figure 2C]. In the 35 EB and LB subjects, SRD thresholds were significantly correlated with the duration of blindness (*r* = 0.386, *p* = 0.024; Figure 3A), whereas TMD thresholds and SRTs were not correlated with duration of blindness (*r* = −0.298, *p* = 0.082 and *r* = −0.104, *p* = 0.552). In 16 LB subjects, SRD thresholds showed a trend toward significance in the correlation with the age at blindness onset (*r* = −0.487, *p* = 0.056; Figure 3B), whereas TMD thresholds and SRTs were not correlated with the age at blindness onset (*r* = 0.297, *p* = 0.264 and *r* = 0.186, and *p* = 0.490). Figure 4 shows the grand mean ACC potentials in response to the ripple noise change at the Cz and Fz electrodes. Although the N1 peak amplitude originating from the inverted ripple sound was numerically greater in EB subjects than in LB subjects or sighted subjects, there were no statistically significant differences [*F*(2:43) = 2.735; *p* = 0.076 and *F*(2:43) = 3.027; *p* = 0.059].

## Discussion

Psychoacoustic data showed that EB subjects showed better performance in auditory spectral and temporal resolution compared with sighted subjects. Our findings are largely consistent with those of previous studies for spectral resolution (Gougoux et al., 2004; Hamilton et al., 2004; Wan et al., 2010), and for temporal resolution (Stevens and Weaver, 2005; Hertrich et al., 2013). Voss and Zatorre (2012) and Voss et al. (2014) demonstrated that heightened pitch discrimination in blind subjects was directly related to the degree of structural neuroplasticity in the cortex. Our behavioral results also support the theory of compensatory plasticity of cerebral cortex following visual deprivation. There are several hypotheses regarding this plasticity. First, the visual cortex is recruited to perform auditory functions (Leclerc et al., 2000; Gougoux et al., 2009; Collignon et al., 2011; Voss and Zatorre, 2012). Second, preexisting audiovisual connections that are masked by a dominant visual input can be unmasked and strengthened (Beer et al., 2011; Collignon et al., 2013; Pelland et al., 2017). Third, new neural connections may develop between each brain region (Karlen et al., 2006; Chabot et al., 2008). On the other hand, another view of no experience of sight in EB subjects proposed that vision is inevitable to develop certain auditory performances in particular with regards to auditory sense of space or auditory map. According to the cross-sensory calibration hypothesis proposed by Gori et al. (2008, 2010, 2014), during early development vision calibrates other senses to process spatial information for which it is the most robust sense. The sound localization task in vertical plane (Zwiers et al., 2001; Lewald, 2002) or the performance of more complex tasks requiring a metric representation of the auditory space (Gori et al., 2010, 2013, 2014; Finocchietti et al., 2015; Vercillo et al., 2016) tend to be worse in EB subjects than in sighted controls. Moreover, a recent study revealed that early occipital activation during spatial bisection tasks was reduced in EB subjects compared with sighted subjects (Campus et al., 2019). We speculate that unlike the sound localization, auditory spectral and temporal resolutions may not require vision for their maturation.

LB subjects showed better temporal resolution, but not spectral resolution, compared with sighted subjects. Two hypotheses are available to explain these results. First, there may be a critical period for plastic changes of spectral resolution at a young age, so LB subjects have limited chance for functional enhancement. In contrast, the better temporal resolution compared with sighted subjects implies that the functional enhancement following visual deprivation might not be affected by the age at onset of blindness. We speculate that this hypothesis is supported by the trends toward significant negative correlations between SRD thresholds and age at blindness onset in LB subjects, whereas TMD thresholds were not correlated with age at blindness onset. We also speculate that the existence of a critical period for plastic changes of spectral resolution is plausible considering the previous reports. Gougoux et al. (2004) reported very similar results to ours, showing that people experiencing blindness at a younger age show better pitch discrimination. Similarly, Voss et al. (2008) found a significant negative correlation between the age at blindness onset and sound-source discrimination. In an fMRI study, Qin et al. (2015) found that visual deprivation before the developmental sensitive period can induce more extensive brain functional reorganization than does visual deprivation after the sensitive period. Second, auditory spectral resolution depends primarily on active movement of outer hair cells and cochlear tuning (Dubno and Schaefer, 1995; Oxenham and Bacon, 2003). However, it has been hypothesized temporal modulations are represented by the neural firing in auditory cortex, and the auditory cortex plays a key role in temporal processing of sound (Schulze and Langner, 1997; Eggermont, 2001; Bao et al., 2004). In support of this temporal coding mechanism, Schnupp et al. (2006) and Bao et al. (2004) reported the plasticity of temporal coding in auditory cortex by sensory training in animals and Asal et al. (2018) reported a similar result for humans. The relatively large contribution of the central auditory system to temporal resolution, and a high degree of plastic property of auditory cortex may induce relatively fast plastic changes in temporal resolution following visual deprivation, and this phenomenon can provide LB subjects with an advantage in temporal resolution compared with sighted subjects, unlike spectral resolution.

In the current study, the EB subjects showed significantly better SRD performance than the LB subjects, but there was no difference in TMD between the two groups. These results may suggest a different pattern or a different speed of functional enhancement following visual deprivation between spectral resolution and temporal resolution. Several hypotheses are available to explain these results. First, the positive significant correlation between SRD and the duration of blindness supports the hypothesis of the gradual improvement of SRD over time following visual deprivation. In contrast, the relationship between TMD and the duration of blindness did not reach statistical significance. Taken together, we speculate that it may take a longer time for auditory spectral resolution to undergo functional enhancement, whereas temporal resolution may improve much more quickly following visual deprivation. Different correlations between the duration of blindness and sound-induced behavioral performances or the sound-related activity of the brain were reported in previous studies. Some authors reported positive correlations (Qin et al., 2015; Tao et al., 2015) whereas others reported negative correlations (Collignon et al., 2013; Wang et al., 2013; Amadeo et al., 2019). Second, several studies reported a natural development of SRD in sighted children with normal hearing threshold showing gradual improvement by 7–11 years of age and adult-like performance was seen at 9–12 years old (Blagosklonova et al., 1989; Allen and Wightman, 1994, Peter et al., 2014). EB subjects go through this developing period without visual stimulation, and therefore this time less than 12 years of age could be a critical period for plastic changes of spectral resolution. In contrast, all LB subjects in the current study have the age of visual loss greater than 12 years of age except for two subjects (9 and 10 years of age). Taken together, it is plausible that if blindness occurs before around the age of 12, such individuals may be able to experience the plastic change of spectral resolution, which may not be manifested in blind subjects whose blindness occur after this critical period. Third, previous studies reported that occipital recruitment for auditory processing appears to be reduced in LB compared with EB (Voss et al., 2008; Bedny et al., 2010, 2012; Collignon et al., 2013). From this perspective, we speculate that the reduced levels of plasticity in LB might have been insufficient to gain an advantage in spectral resolution in the current study, but it could be still sufficient to gain an advantage in temporal resolution.

Although EB and LB subjects showed advantages in auditory spectral and temporal resolution compared with sighted controls, we found no significant differences in speech perception in noise among the three groups. Only a few studies have examined the speech perception ability in a similar setting, and their results were inconsistent with each other (Muchnik et al., 1991; Gougoux et al., 2009; Arnaud et al., 2018). Muchnik et al. (1991) found no difference in speech perception in silent conditions between a mixed group of EB and LB subjects and a group of sighted subjects, but the blind subjects outperformed the sighted subjects when tested in noisy conditions. Gougoux et al. (2009) found no behavioral differences in voice recognition among EB, LB, and sighted control subjects. Arnaud et al. (2018) demonstrated better discrimination for native vowel in the blind subjects compared with sighted subjects, but no difference for non-native vowel. In this study, two-syllable words were presented in the presence of background noise. As such, the listener’s ability to understand semantic cues might play an important role in addition to the ability to discriminate subtle speech cues. It is possible that speech discrimination measures that isolate semantic cues and force listeners to focus on acoustic cues for discrimination may better characterize the difference in speech understanding between EB subjects and sighted subjects. Further studies are therefore needed to assess speech perception among blind subjects.

There are two limitations in the present study. Although we analyzed the data using ANCOVA to control for differences in age, the fact that younger age of sighted subjects compared with the LB subjects might have affected the result of temporal resolution because sighted subjects had a less amount of experience compared with LB subjects. The second one is that age at blindness onset for the LB group was quite variable. However, this is unlikely to affect the results for the following reasons. Earliest onset of blindness was at 9 years of age, and therefore, a significant amount of time has passed since their birth. Furthermore, in 16 LB subjects, the three psychoacoustic performance measures (i.e., SRD thresholds, TMD thresholds, and SRTs) were not correlated with the age at onset of blindness.

## Conclusion

In conclusion, compared with sighted subjects, EB subjects showed advantages in auditory spectral and temporal resolution, while LB subjects showed an advantage in temporal resolution exclusively. These findings suggest that it might take longer for auditory spectral resolution to functionally enhance following visual deprivation compared with temporal resolution or a critical period of very young age is required to improve auditory spectral resolution following visual deprivation.

## Data Availability Statement

All datasets generated for this study are included in the article/supplementary material.

## Ethics Statement

The studies involving human participants were reviewed and approved by the Institutional Review Board of Eulji Medical Center, Seoul, South Korea. The patients/participants provided their written informed consent to participate in this study.

## Author Contributions

HS: conceptualization. GG, HL, SC, and JW: methodology. GG: investigation. GG and HL: data analysis. HS: writing original draft. JW: writing review and editing.

## Conflict of Interest

The authors declare that the research was conducted in the absence of any commercial or financial relationships that could be construed as a potential conflict of interest. The views expressed in this paper are those of the authors and do not necessarily reflect the official policy or position of the US Department of Health and Human Services and the U.S. Food and Drug Administration. No official endorsement is intended or should be inferred.
